# Refining our understanding of the diversity of plant specialised metabolites

**DOI:** 10.1111/nph.20173

**Published:** 2024-10-09

**Authors:** Mike Speed, Graeme Ruxton

**Affiliations:** ^1^ School of Biosciences, Faculty of Health & Life Sciences University of Liverpool Liverpool UK; ^2^ School of Biology University of St Andrews St Andrews UK

**Keywords:** coevolution, evolvability, herbivory, plant biochemistry, plant–animal interactions

## Abstract

This article is a Commentary on Wittmann & Bräutigam (2025), **245**: 1302–1314.

At some levels of organisation, plants seem simple compared with animals – having only two systems (root and shoot), three types of tissue (dermal, vascular, and ground) and four types of organs (leaves, stems, roots, and sometimes reproductive organs). However, biochemically, plants seem very complex. About 8000 so‐called primary metabolites are shared across all plants and can be shown to be vital to growth, development or reproduction. On top of that, there are what were previously called secondary metabolites, but are increasingly called specialised metabolites. These compounds are not found universally across plants and have roles that are not essential to vital functions but rather are linked to a plant's environmental responses. The environment an organism experiences can be unpredictable and can change multiple times in its lifetime. For animals, some responses to environmental challenges are biochemical (think of our immune system), but movement and behaviour are particularly important. With plants lacking the ability to escape enemies, biochemistry is key to protection – and so we could perhaps expect plants to have more diverse specialised metabolites than animals – but the diversity is amazing. We have not counted all the plant specialised metabolites, but estimates range from 200 000 to 1000 000 (Jacobowitz & Weng, 2020; Weng *et al*., 2021; Huang & Dudareva, 2023). Nicotine is a very well‐known plant specialised metabolite that functions to deter herbivorous insects, but it occurs in only one family of plants (the nightshades) and varies greatly in concentration – being found in high concentrations in tobacco but at only trace levels in potatoes and tomatoes. In an article published in this issue of *New Phytologist*, Wittmann & Bräutigam (2025; pp. 1302–1314) offer a new mathematical modelling approach to explain this diversity.

‘…the work of Wittmann & Bräutigam offers useful and testable predictions on a number of hypothesised mechanisms for the diversity of plant specialised metabolites…’

Of course, plant chemodiversity has been addressed before (e.g. Wetzel & Whitehead, 2020), including through quantitative modelling (Thon *et al*., 2024). The current study provides an important contribution by taking a simple but broadly applicable population‐genetic modelling approach that allows analytic predictions to complement the simulation study, and exploring the validity of five explanations within this common framework. For each of these hypotheses, they offer valuable insights.

Wittmann & Bräutigam give the most emphasis to the recently highlighted ‘dominance reversal hypothesis’ (Grieshop *et al*., 2024), likely because their explicitly genetic model is ideally suited to exploring this idea. Simply put, this hypothesis suggests that diversity will be maintained (through heterozygote advantage) if alleles conferring a metabolite defensive against an antagonist are dominant with respect to benefits but recessive with respect to costs. Their model predicts that such complete reversal is not required; rather, polymorphism will be maintained provided that the degree of dominance is sufficiently different between the benefits and costs that heterozygotes experience with relatively high benefits and low costs. This refinement of the hypothesis is important because we see no *a priori* reason to expect that dominance relationships are necessarily identical across pleiotropic phenotypes; inequalities could be common.

Another important explanation for chemodiversity is termed the ‘fluctuating‐selection hypothesis’ by Wittmann & Bräutigam. Here, heterozygotes have the highest geometric mean fitness over time because of temporal variation in the selective environment, which is sufficiently strong against the phenotypes of the respective homozygotes. The obvious example driver of a changing selective environment would be a trait that offers defence against one herbivore type but actually facilitates another herbivore type when there is strong variation in the relative challenge from these two herbivores over time. The authors found that this hypothesis was logically consistent, and polymorphism could be maintained by this mechanism, but effect sizes were modest. They argue that many types of fluctuation in a selective regime can even out over the timescales relevant to evolutionary change in the plant population.

Perhaps the most obvious explanation for metabolite diversity is that the environment a plant might have to contend with may vary in a highly multidimensional space. For example, plants might need a diversity of different defences against potential threats from a taxonomically diverse array of potential herbivores. But even in this case, Wittmann & Bräutigam offer interesting insights. They found that increasing the diversity of herbivore challenges in their model selected for an increase in the average number of metabolites per individual. Interestingly, the total number of metabolites expressed across all individuals in the population did not increase, potentially because there was a higher probability of loss of rare alleles in situations with diverse herbivore challenges. Increased herbivore diversity also led to increased metabolite diversity as measured by the increased differences in the metabolic compositions of two random plant individuals drawn from the population. The key message here is that there are a number of biologically meaningful ways of describing the diversity of metabolites at individual and population levels, and different candidate mechanisms will have differing effects on these different measures. Thus, considering different types of diversity may be important to developing an understanding of the relative importance of different underlying driving mechanisms.

Wittmann & Bräutigam's model often assumes that protection increases with the quantity of toxins, and hence, a driver of chemodiversity is a greater concentration of metabolites per se. However, they also considered synergy between metabolites, where combinations of specific compounds can have disproportionately effective defensive outcomes. Here, their model predicted (unsurprisingly) that where different metabolites work synergistically, the stronger such synergistic effects are, the higher the number of metabolites per individual, and across the whole population. However, diversity, as measured by the average number of differences in metabolite composition between two random individuals in the population, decreases. This is an inevitable consequence of more individuals in the population carrying alleles for more of the suite of possible metabolites; however, this again emphasises that how we measure diversity really matters.

Lastly, Wittmann & Bräutigam explore the ‘screening’ hypothesis that suggests that a novel metabolite has a low probability of offering a benefit to the individual plant in its current environment, so plants are forced to produce a large number of different metabolites in order to generate a few metabolites that actually are useful. They offer model predictions that are consistent with the hypothesis but concede that their model structure is not ideal for the evaluation of this hypothesis, which may require a systems‐simulation approach. This leads us to how future work could build on the methodology of Wittmann & Bräutigam.

At the empirical level, this paper will, we hope, be a stimulus for researchers to investigate the dominance characteristics of traits that are under antagonistic pleiotropy, trading off defence against herbivores with, for example, reproductive output. Wittmann & Bräutigam do well, in our view, to bring this to the attention of those who seek explanations for chemodiversity in plants; as they point out, the knowledge base here is very small.

Finding the right balance of model complexity is the key dark art of theoretical biology. With this in mind, we tentatively suggest including ecological and physiological aspects to add complexity to Wittmann & Bräutigam's framework in order to offer a more rigorous evaluation of a greater range of drivers of metabolite diversity.

Ecologically, a considerable limitation of the current framework of Wittmann & Bräutigam is that the environment (the herbivore challenge) does not change in response to the simulated evolution of the plant population under pressure from those herbivores. In reality, we would often expect coevolution, and exploration of the consequences of this has already begun (Speed *et al*., 2015). However, even on shorter timescales, we might expect the herbivore challenge to one plant to be a function not only of its defences but also of other plants in the same population – and even plants of different species in the local vicinity, and again, there is existing work that could usefully be integrated into the population‐genetics framework here (Underwood *et al*., 2014; Sato, 2018).

Going in the physiological direction, Wittmann & Bräutigam model a very simple linkage between genotype and phenotype, but in reality, we know that there are sources of complexity, shared pathways of synthesis, and competition for resources will mean that the expression of some metabolites will facilitate or inhibit the expression of others. Allowing for more careful modelling of the physiology of metabolite expression will allow more subtle (but testable) predictions about the nature of the metabolite portfolios shown by plants, and which metabolites are environmentally induced and which are more constitutive. Accepting that the expression of a single metabolite will not be under the control of a single dedicated gene will also allow the exploration of the evolvability of plant specialised metabolites and allow us to make predictions about; which of a range of responses to a novel environmental challenge might most readily evolve; on what timescale; and what the costs of being able to respond quickly and flexibility are. Again, there is a body of work to build on in this respect (Ono & Murata, 2023).

In summary, the work of Wittmann & Bräutigam offers useful and testable predictions on a number of hypothesised mechanisms for the diversity of plant specialised metabolites. It offers a powerful, general and flexible modelling framework that could be developed further to offer even richer predictions. However, potentially, the most immediately beneficial outcome of this work is the emphasis that there are a range of interconnected but fundamentally different measures of diversity. This should encourage us to ask more subtle questions than ‘Why is there so much diversity?’ and focus more on attempting to describe (and then explain) the nature of the diversity that we see in different plant individuals, populations, species, and families.
